# Persistence of COVID-19 Symptoms and Quality of Life at Three and Twelve Months after Hospital Discharge

**DOI:** 10.3390/medicina60060944

**Published:** 2024-06-05

**Authors:** Lizeth Guadalupe Gutiérrez-Canales, Carolina Muñoz-Corona, Isaac Barrera-Chávez, Carlos Viloria-Álvarez, Alejandro E. Macías, Liz Jovanna Martínez-Navarro, Jose A. Alvarez, David Alejandro Scavo-Montes, Eduardo Guaní-Guerra

**Affiliations:** 1 General Directorate of Quality and Health Education, Ministry of Health, Ciudad de Mexico 11410, Mexico; lg.gutierrezcanales@gmail.com; 2 Department of Medicine, University of Guanajuato, León 37670, Guanajuato, Mexico; itzatcarolina07@gmail.com (C.M.-C.); id.barrerachavez@ugto.mx (I.B.-C.); jcviloria1809@gmail.com (C.V.-Á.); aaeemmhh@yahoo.com (A.E.M.); alvarez_ja@me.com (J.A.A.); 3 Servicios de Salud del Instituto Mexicano del Seguro Social Para el Bienestar (IMSS-BIENESTAR), Hospital Regional de Alta Especialidad del Bajío, León 37544, Guanajuato, Mexico; falcot23@hotmail.com; 4 Hospital Estatal de Atención COVID-19, León 37000, Guanajuato, Mexico; davidscavomontes@gmail.com

**Keywords:** quality of life, coronavirus disease 2019, SARS-CoV-2, 36-item Short-Form Survey (SF-36), persistent COVID-19 symptoms, long-COVID

## Abstract

*Background and Objectives:* Medical and public recognition of “long-COVID or post-COVID syndrome”, as well as its impact on the quality of life (QoL), is required to better address the disease burden. Objectives: We aimed to describe the persistence of COVID-19 symptoms and QoL among patients at three and twelve months after their discharge from the hospital. *Materials and Methods:* We conducted an observational, prospective, and longitudinal analytic study from September 2021 to April 2022. To measure QoL, we used a validated version of the 36-item Short-Form Health Survey (SF-36). *Results:* We included 68 patients in the study. A total of 54 (79.4%) patients reported at least one persistent symptom at three months vs. 52 (76.4%) at twelve months (*p* = 0.804). Some persistent symptoms (myalgia, alopecia, and cough) decreased significantly at twelve months (50% vs. 30.9%, 29.4% vs. 13.2%, and 23.5% vs. 7.4%; respectively, *p* = 0.007); in contrast, other persistent symptoms (sleep–wake and memory disorders) were more frequent (5.9% vs. 32.4% and 4.4% vs. 20.6%; respectively, *p* = ≤0.001). Regarding QoL, a statistically significant improvement was observed in some scores over time, *p* = ≤0.037. At twelve months, dyspnea, myalgia, and depression were risk factors associated with a poor physical component summary (PCS), *p* = ≤0.027, whereas anxiety, depression, and fatigue were associated with a poor mental component summary (MCS), *p* = ≤0.015. *Conclusion:* As the proportion of persistent symptoms at twelve months is high, we suggest that patients must continue under long-term follow up to reclassify, diagnose, and treat new onset symptoms/diseases.

## 1. Introduction

In addition to more than 770 million cases and 6.9 million deaths [1], COVID-19 has had other important consequences, one of which is the recent concept of “long-COVID” or “post-COVID syndrome”, which some experts describe as the persistence of symptoms after SARS-CoV-2 infection [2]. The pathogenesis of long-COVID syndrome is poorly understood, but a major contributory mechanistic factor is the persistent cytokine storm that may last longer in long-COVID patients than in others, probably triggered by aggregates of SARS-Co-2 discovered recently in the adrenal cortex, kidney, and brain [3].

The symptoms can affect several body systems, including the pulmonary, cardiovascular, and nervous system, in addition to psychological effects. In October 2021, the World Health Organization (WHO) recognized the existence of a post-COVID condition through a Delphi consensus, acknowledged the challenge of its diagnosis and treatment, and allowed for possible changes in its definition [4,5]. Faghy et al. mentioned the importance of medical and public recognition of disabilities associated with long-COVID; likewise, urgent research is required to develop diagnosis, treatment, and support mechanisms that can better address the growing and future burden of long-COVID [5]. 

In association with the recognition of the effects of the long-COVID, we consider that evaluation of the quality of life (QoL) is important to visualize the patients’ statuses in relation to long-COVID effects. We previously evaluated the relationship between persistent long-COVID symptoms and QoL three months after hospital discharge, observing that patients with persistent symptoms had lower QoL (SF-36) scores than patients without symptoms [6]. Qu et al. also observed in their study that the presence of persistent COVID-19 symptoms was associated with poor QoL, specifically poor physical component summary (PCS), at three months after discharge [7]. However, patients with persistent symptoms and diminished QoL due to these symptoms need to be followed up for a longer period to observe whether their persistent symptoms continue or disappear. 

We aimed to describe the persistent symptoms and QoL of individuals at three and twelve months after hospital discharge for COVID-19, as well as to determine the risk factors associated with decreased QoL at twelve months. 

## 2. Materials and Methods

### 2.1. Study Design

We conducted an observational, prospective, and longitudinal analytic study at two COVID-19 referral centers (Hospital Regional de Alta Especialidad del Bajío and Hospital Estatal de Atención COVID-19) in the state of Guanajuato, Mexico. The questionnaires were applied from August 2020 to April 2022. This study was conducted in accordance with Strengthening the Reporting of Observational Studies in Epidemiology (STROBE) guidelines [8]. 

### 2.2. Study Population

We included men and women ≥ 18 years old with confirmed diagnosis of COVID-19 by RT-PCR who required hospitalization due to SARS-CoV-2 infection. Exclusion criteria were patients who did not give informed consent and those who did not answer the second questionnaire at twelve months. After a twelve-month follow-up period, only 68 patients of the 141 that met the inclusion criteria at three months completed the interview and questionnaire and were included in the study. At twelve months after discharge, all patients had had at least two SARS-CoV-2 vaccines.

### 2.3. Study Outcomes

Primary outcomes: Persistence of symptoms and QoL at three and twelve months were assessed by an interview and a standard questionnaire. QoL was assessed by the SF-36 survey at three and twelve months after hospital discharge for SARS-CoV-2 infection. Total scores ranged from 0 to 100, with a higher score indicating a better outcome.

Secondary outcomes: risk factors associated with poor health-related quality of life (HRQoL) at twelve months. Poor HRQoL was defined as scores of PCS or MCS less than 50.

### 2.4. Study Instruments

Epidemiological and clinical data were obtained by reviewing clinical records. The authors interviewed patients and established a prospective database using a standard questionnaire to obtain general characteristics (days between the onset of symptoms and hospitalization, days since discharge, home implemental oxygen, complications and sequelae attributed to COVID-19) and persistent symptoms at three and twelve months after discharge (fatigue, dyspnea, joint pain, chest pain, cough, anosmia, headache, sicca syndrome, red eyes, sputum production, lack of appetite, sore throat, vertigo, myalgia, diarrhea, anxiety, depression, memory disorders, sleep–wake disorders, neuromuscular diseases, and alopecia). Depression and anxiety were measured using a validated Spanish version of the nine-item Patient Health Questionnaire depression scale (PHQ-9) and seven-item Generalized Anxiety Disorder scale (GAD-7) [9,10]. To measure QoL, we used a validated version of the MOS/RAND 36-item Short-Form Health Survey (SF-36) in Spanish for the Mexican population [11,12,13]. The SF-36 questionnaire consists of 36 questions grouped into eight dimensions: physical functioning (PF, limitations in performing physical activities such as bathing or dressing), role physical (RP, limitations in work and other daily activities as a consequence of physical health), bodily pain (BP, how severe and limiting is the pain), general health (GH, how patients perceive their current personal health), vitality (VT, feeling tired and worn out as opposed to feeling energetic), social functioning (SF, how physical or emotional problems intervene with social activities), role emotional (RE, limitations in work and other daily activities as a result of emotional problems), and mental health (MH, feeling nervous and depressed as opposed to peaceful, happy, calm, or quiet). We included the health change score (HC, change in overall health status from last year). The scores for each item ranged from 0 to 100, with higher scores indicating better health status. Additional scores were calculated using the physical component summary (PCS) and mental component summary (MCS), along with the use of the oblique (correlated) method proposed by Ware and Hays, the creators of these scores [14]. The PCS was derived from positive weighting for the PF, RP, BP, GH, and VT domains and negative weighting for the SF, RE, and MH domains. The MCS was derived from positive weighting for the VT, SF, RE, and MH domains and negative weighting for the PF, RP, BP, and GH domains. The MCS and PCS are presented as T-scores [15].

### 2.5. Ethics

The study was approved by the Ethics and Research Committee of the Hospital Regional de Alta Especialidad del Bajío (approval number CEI-26-2020/date of approval 29 June 2020 and CEI-25-2021/date of approval 12 July 2021). Informed consent was obtained from all participants. 

### 2.6. Statistical Analysis

Quantitative variables are described using mean ± standard deviation or median with interquartile range (IQR). Categorical data are described using absolute and relative frequencies. Normal distribution was assessed for continuous variables using the Kolmogorov–Smirnov test. In a comparative analysis among SF-36 scores over time (three and twelve months), data were compared using the Wilcoxon signed-rank test. Statistical differences between study groups (persistence of symptoms three vs. twelve months after hospital discharge due to COVID-19) were calculated using the McNemar test. The odds ratio (OR) and 95% confidence interval (95% CI) were calculated for qualitative dichotomous variables. Statistical analysis was performed using VassarStats [16]. A *p*-value of <0.05 was considered significant. Univariable and multivariable logistic regression models were used to explore risk factors associated with poor health-related quality of life (HRQoL). All variables that were significant in the univariate logistic regression models were included in the multivariable logistic regression model performed by hierarchical selection. Poor HRQoL was defined as scores of PCS or MCS less than 50, as previously described in other studies [17,18]. The logistic regression analyses were performed with NCSS 12.0.2 Statistical Software (NCSS, LLC, Kaysville, UT, USA, 2018).

## 3. Results

A total of 68 patients complied with the inclusion criteria and were evaluated at three and twelve months after discharge due to COVID-19. The male-to-female ratio was 1.6 to 1. The mean patient age at the beginning of the study was 50.93 ± 14.98 years. At the time of the study, 82.4% patients had associated chronic comorbidities. See Table 1. 

Concerning the persistence of COVID-19 symptoms, 54 (79.4%) patients reported at least 1 persistent symptom at three months vs. 52 (76.4%) at twelve months (*p* = 0.804; OR = 1.87, 95% CI: 0.53–2.67). Among those with persistent symptoms at twelve months, 8 (11.76%) patients had only one symptom, 9 (13.23%) had two, and 40 (58.82%) had three or more. The main persistent symptoms at twelve months were fatigue (35) (51.1%) and joint pain (30) (44.1%).

We observed that myalgia, alopecia, cough, and sputum production decreased significantly by twelve months. Other persistent symptoms tended to decrease over time, but no statistical significance was found. In contrast, sleep–wake and memory disorders increased significantly at twelve months. See Table 2. 

Regarding the SF-36 questionnaire, data analysis showed that at twelve months, the most affected domain was general health (GH) (median score 70; IQR 60–85); in contrast, the most affected domain at three months was health change (median score 37.50; IQR 25–50). A statistically significant improvement was observed for different scores (PF, RP, GH, HC, PCSc and MCSc) over time. See Table 3.

In the multivariate logistic regression model, some factors associated with poor PCS and MCS were found. The results indicated that some persistent symptoms, such as dyspnea, myalgia, and depression, were risk factors for poor PCS. On the other hand, having anxiety and depression was associated with poor MCS. See Table 4.

## 4. Discussion

Our study found a similar percentage of at least one persistent COVID-19 symptom at three and twelve months after hospital discharge (79.4% vs. 76.4%; *p* = 0.804). The main persistent symptoms at twelve months were fatigue and joint pain. Regarding the QoL, the PF, RP, GH, HC, PCSc, and MCSc domains of the SF-36 questionnaire increased their scores over time. At twelve months, dyspnea, myalgia, and depression were risk factors associated with poor PCSc; anxiety, depression and fatigue were risk factors for poor MCSc.

As mentioned before, the most persistent symptom in our patients at twelve months was fatigue (51.1%). Likewise, Brunvoll et al. and Evans et al. also found fatigue as the most persistent symptom (30.6% and 49.9%) in their respective studies at 15 months and 5.9 months after COVID-19 [19,20]. Crook et al. considered fatigue as a major manifestation of long-COVID, where systemic inflammation; cell-mediated immune mechanisms; or even direct SARS-CoV-2 infection of the skeletal muscle resulting in damage, weakness, and inflammation to muscle fibers and neuromuscular junctions could be involved in the physiopathogenesis [21]. Finsterer et al. considered that fatigue can be a subjective sensation or an objective, measurable phenomenon called fatigability. Therefore, they proposed an objective measure of fatigability with a maximal voluntary contraction or peak force that can be measured with a dynamometer or electromyography [22]. Further studies should be conducted to assess fatigue objectively in patients who have had COVID-19 and suffered long-COVID, as well as to find the mechanisms implicated with this symptom. 

Regarding joint pain, the second most persistent COVID-19 symptom in our study (44.1%), the observed proportion was similar to that reported by Evans et al. and Gamberini et al., with this symptom affecting 47.8% and 34.8% of their studied patients after a follow-up of 5.9 and 12 months, respectively [20,23]. Baimukhamedov Ch. has previously suggested that, since COVID-19 may be a trigger of rheumatic diseases, if symptoms persist longer than twelve months, a thorough review for diagnosis of new-onset rheumatoid musculoskeletal disease would be advisable [24]. We agree with this approach and recommend a longer follow-up period for patients with joint pain at twelve months in order to search for, reclassify, or diagnose a new-onset rheumatoid musculoskeletal disease.

Interestingly, some persistent symptoms decreased significantly from three to twelve months, like myalgia, alopecia, cough, and sputum production. Myalgia decreased from 50% to 30.9% in our study group at twelve months (*p* < 0.05). This trend has been reported by Evans et al. and Gamberini et al. in separate studies, as they observed myalgia in 57.2% and 34.8% of their patients at 5.9 months and 12 months, respectively, after discharge for COVID-19 [20,23]. Another symptom that decreased over the time was alopecia, as it was observed in 29.4% patients at three months vs. 13.2% at twelve months (*p* < 0.05). The reason why the frequency of persistent COVID-19 symptoms decreases eventually could be related to diminished viral persistence in some organs and systems over time, as well as recovery of the functionality of T lymphocytes that lost their ability to react during SARS-CoV-2 infection. It could even be associated with less pandemic stress, as has been proposed for alopecia [25,26,27]. The fact that most persistent COVID-19 symptoms decrease over time is encouraging for the improvement of patients’ QoL, as well as the return to the life they had before COVID-19. 

In contrast, some persistent COVID-19 symptoms increased in frequency from three to twelve months. Regarding memory disorders, these were augmented from 4.4% to 20.6% (*p* < 0.01) at 3 and 12 months, respectively. Kim et al. mentioned in their study that the most frequent persistent symptoms at twelve months were amnesia (24.1%) and insomnia (14.7%), which is consistent with our findings [28]. Concerning this, Xu. et al. found a hazard ratios (HRs) of 1.77 and 20.03 for cognition/memory disorders and Alzheimer´s Disease, respectively, at twelve months after COVID-19 [29]. Golzari-Sorkeh et al. reviewed some pathological mechanisms involved with cognitive memory disorders and Alzheimer´s Disease after COVID-19 [30]. One of these mechanisms is the deposition of amyloid ß (Aß) during the COVID-19 pro-inflammatory cytokine storm, as the microglial cells lose their capacity to effectively phagocytose Aß [30]. Monje et al. mentioned that neurological and neuropsychiatric symptoms affect a substantial fraction of people after COVID-19; this decreases the QoL and leads to an inability to return to previous levels of occupational function [31]. We consider memory disorders, including Alzheimer´s Disease, to be serious chronic conditions that will impact some people for the rest of their lives, so it is important to evaluate and perform timely diagnoses in those patients who report memory and cognitive problems, as the risk of developing Alzheimer´s Disease after COVID-19 is high.

Regarding sleep–wake disorders, we observed an increase from 5.9% to 32.4% (*p* < 0.01) when comparing the patients at 3 vs. 12 months. This is similar to data reported by Formiga et al., where 25.3% of patients had sleep–wake-related symptoms eighteen months after SARS-CoV-2 infection [32]. The possible implicated mechanisms are direct viral invasion, prolonged latent inflammation, and immunosenescence of T-cells [32]. However, Crook et al. consider that stress, anxiety, and other negative emotions must be associated with sleepiness [21]. Given the health impact of sleep disorders, it would be advisable for patients to receive professional care from specialists in these pathologies to improve their quality of sleep, performance, and emotional problems. 

Concerning QoL, in our study, the main parameter affected at twelve months was GH, with a score of 70 (IQR 60–85), which is consistent with findings reported by Eberst et al., who found a GH score of 65 at twelve months after COVID-19 discharge [33]. Characteristically, we observed statistically significant increments in PF, RP, GH, HC, PCS, and MCS scores from three to twelve months. This is consistent with a QoL study published by Wang et al. in a healthy population from Shanghai, since we found similar SF-36 scores among their healthy subjects and our patients at twelve months [34]. 

Regarding MCS and PCS scores, O´Kelly reported that both scores increased from three months to twelve months with statistical significance, which is in accordance with our observations; however, they used the SF-12 questionnaire and did not mention the method they used to obtain PCS and MCS scores [35]. These findings are encouraging, as the QoL of patients who suffered SARS-CoV-2 infection and have long-COVID may improve over time, to the point of being comparable to that of healthy people. 

As mentioned previously, some factors associated with poor PCS and MCS were found. Persistence of dyspnea, myalgia, and depression were associated risk factors with poor PCS. This is consistent with the findings we previously reported, as well as those observed by Qu et al., in which the presence of persistent COVID-19 symptoms was associated with poor PCS [7,36]. On the other hand, having anxiety, depression, and fatigue was associated with poor MCS. This is, again, comparable with the findings of Gutierrez et al., as they observed that having depression was a risk factor for poor MCS. To date, it is not clear whether depression and anxiety are directly caused by SARS-CoV-2 infection. Certainly, some factors related to the COVID-19 pandemic, like quarantine, fear of SARS-CoV-2 infection, and overexposure to COVID-19-related news, could have impacted the population in terms of mental health, causing a co-occurring psychiatric epidemic. Also, anxiety and depression could have been undiagnosed comorbidities present in patients prior to COVID-19, and may have been misdiagnosed [7]. On the other hand, patients who had COVID-19 developed higher levels of anxiety, depression, and post-traumatic stress compared to non-COVID controls, suggesting a direct role of SARS-CoV-2 in the development of those symptoms [37]. Regardless of the cause, a prompt diagnosis and timely intervention would be important, since QoL can be significantly improved in patients with depression and anxiety.

Our study has some limitations. One of them was the sample size; this could decrease the power of the study and may not reflect the entire population. In addition, we did not include healthy controls to analyze whether QoL at twelve months was comparable to that of completely healthy patients. We did not assess the health status of the patients prior to COVID-19, and we did not know whether some of these symptoms were present prior to SARS-CoV-2 infection and hospitalization. However, despite this, the study has one great strength: We followed the same group of patients over time and observed improvement or worsening of the persistence of symptoms and quality of life. 

## 5. Conclusions

We found that the percentage of at least one persistent symptom of COVID-19 at three and twelve months after hospital discharge was similar and remained high. The main persistent symptoms at twelve months were fatigue and joint pain. Some persistent symptoms tended to decrease from three to twelve months, like myalgia, alopecia, cough, and sputum production; on the other hand, some persistent symptoms tended to increase over time, like sleep–wake disorders and memory disorders. 

Regarding the SF-36 of the QoL questionnaire, the PF, RP, GH, HC, PCS, and MCS domains increased in their scores significantly from three to twelve months. At twelve months, dyspnea, myalgia, and depression were risk factors associated with poor PCS, whereas anxiety, depression, and fatigue were associated with poor MCS. 

As the proportion of persistent symptoms at twelve months is high, even when the QoL tend to improve in most patients, we suggest that patients that remain symptomatic continue under long-term follow up in a long-COVID clinic or with trained healthcare personnel to reclassify, diagnose, and treat new-onset symptoms/diseases based on the new guidelines as we continue learning about COVID-19 and its repercussions on global health. 

## Figures and Tables

**Table 1:** General characteristics of the study population (*n* = 68).

Characteristics	Value
Age (years), mean (±SD)	50.93 (±14.98)
Male sex, *n* (%)	42 (61.8%)
Female sex, *n* (%)	26 (38.2%)
Smoking, *n* (%)	23 (33.8%)
Comorbidities, *n* (%)	56 (82.4%)
Diabetes, *n* (%)	19 (27.9%)
Hypertension, *n* (%)	31 (45.6%)
Overweight, *n* (%)	24 (35.3%)
Chronic kidney disease, *n* (%)	3 (4.4%)
Chronic obstructive pulmonary disease, *n* (%)	3 (4.4%)
Cardiopathy, *n* (%)	4 (5.9%)
Dyslipidemia, *n* (%)	6 (8.8%)
Asthma, *n* (%)	3 (4.4%)
Cancer, *n* (%)	3 (4.4%)
Other comorbidities, *n* (%)	11 (16.2%)
Days between the onset of symptoms and hospitalization, mean (±SD)	8.31 (±3.82)
Days since discharge, median (IQR)Days since symptoms onset, median (IQR)	416.50 (374.25–453) 422.5 (383–462.75)
Length of hospital stay in days, median (IQR)	8 (5–18.75)
Intensive care unit/mechanical ventilation, *n* (%)	23 (33.8%)
Number of days with mechanical ventilation, mean (±SD)	10 (±7.72)
Complications/sequelae, *n* (%)	63 (92.6%)
Home implemental oxygen, *n* (%)	1 (1.5%)
Critical illness neuropathy/myopathy, *n* (%)	13 (19.1%)
Myocardial infarction, *n* (%)	2 (2.9%)

**Table 2:** Persistent symptoms three and twelve months after hospital discharge due to COVID-19.

	Group (*n* = 68)		*p*-Value *	OR (CI 95%)
	3 Months	12 Months		
Persistent symptoms, *n* (%)	52 (76.5)	54 (79.4)	0.804	1.87 (0.53–2.67)
Fatigue, *n* (%)	38 (55.9)	35 (51.5)	0.648	0.84 (0.43–1.64)
Joint pain, *n* (%)	33 (48.5)	30 (44.1)	0.664	0.83 (0.43–1.66)
Dyspnea, *n* (%)	33 (48.5)	25 (36.8)	0.152	0.62 (0.31–1.22)
Anxiety, *n* (%)	29 (42.6)	25 (36.8)	0.454	0.73 (0.39–1.55)
Sleep–wake disorders, *n* (%)	4 (5.9)	22 (32.4)	0.000	7.65 (2.47–23.70)
Myalgia, *n* (%)	34 (50)	21 (30.9)	0.007	0.45 (0.22–0.90)
Headache, *n* (%)	21 (30.9)	21 (30.9)	1.000	1.00 (0.48–2.07)
Depression, *n* (%)	19 (27.9)	16 (23.5)	0.549	0.79 (0.37–1.71)
Memory disorders, *n* (%)	3 (4.4)	14 (20.6)	0.001	5.61 (1.53–20.57
Chest pain, *n* (%)	12 (17.6)	13 (19.1)	1.000	1.10 (0.46–2.62)
Neuromuscular diseases, *n* (%)	6 (8.8)	11 (16.2)	0.302	1.99 (0.69–5.74)
Vertigo, *n* (%)	9 (13.2)	10 (14.7)	1.000	1.13 (0.42–2.98)
Sicca syndrome, *n* (%)	18 (26.5)	9 (13.2)	0.049	0.42 (0.17–1.02)
Alopecia, *n* (%)	20 (29.4)	9 (13.2)	0.007	0.36 (0.15–0.87)
Red eyes, *n* (%)	5 (7.4)	8 (11.8)	0.453	1.68 (0.52–5.42)
Sputum production, *n* (%)	19 (27.9)	7 (10.3)	0.008	0.29 (0.11–0.76)
Cough, *n* (%)	16 (23.5)	5 (7.4)	0.007	0.25 (0.09–0.75)
Lack of appetite, *n* (%)	5 (7.4)	4 (6.3)	1.000	1.00 (0.27–3.62)
Sore throat, *n* (%)	11 (16.2)	4 (5.9)	0.092	0.32 (0.98–1.07)
Diarrhea, *n* (%)	4 (5.9)	3 (4.4)	1.000	0.73 (0.16–3.43)
Anosmia, *n* (%)	8 (11.8)	3 (4.4)	0.180	0.35 (0.08–1.36)

* Statistical significance calculated using McNemar test. COVID-19, coronavirus disease 2019; OR odds ratio.

**Table 3:** SF-36 total scores three and twelve months after hospital discharge due to COVID-19.

SF-36 Outcomes	Group (*n* = 68)		*p*-Value *
	3 Months	12 Months	
	Score, Median (IQR)	Score, Median (IQR)	
Physical function (PF)	80 (50–90)	85 (65–95)	0.003
Role physical function (RP)	37.50 (0–75)	75 (25–100)	0.000
Body pain (BP)	78 (45.75–100)	85 (58.50–100)	0.259
General health (GH)	67.50 (40–80)	70 (60–85)	0.002
Mental health (MH)	76 (60–87)	76 (64–88)	0.211
Role emotional (RE)	100 (0–100)	100 (41.23–100)	0.103
Vitality (VT)	67.50 (55–85)	75 (56.25–85)	0.286
Social function (SF)	75 (50–100)	75 (62.50–100)	0.095
Health change (HC)	37.50 (25–50)	75 (50–75)	0.000
PCSc	45.10 (37.86–49.91)	49.41 (41.89–55.23)	0.000
MCSc	49.55 (39.65–55.10)	51.99 (44.16–57.36)	0.037

PCSc, correlated physical component summary; MCSc, correlated mental component summary. * Statistical significance was calculated using the Wilcoxon signed-rank test.

**Table 4:** Results of multivariate logistic regression for HRQL of COVID-19 patients.

Outcomes	Variables	Beta	Odds Ratio (95% CI)	*p*-Value
Poor PCSc	Dyspnea	1.450	4.265 (1.18–15.37)	0.027
	Myalgia	1.637	5.142 (1.32–20.08)	0.018
	Depression	3.426	30.751 (3.35–281.84)	0.002
Poor MCSc	Anxiety	1.908	6.740 (1.45–31.26)	0.015
	Depression	3.456	31.685 (3.06–328.07)	0.004
	Fatigue	1.44	4.23 (0.87–20.65)	0.074

PCSc, correlated physical component summary; MCSc, correlated mental component summary.

## Data Availability

All relevant data are within the paper.
